# ID 236 - Análise da Qualidade Metodológica de Ensaios Clínicos sobre o Uso da Eritropoetina Comparado à Hidroxiureia em Pacientes com Anemia Falciforme

**DOI:** 10.5327/2237-9622.2025.v34s1.236

**Published:** 2025-11-25

**Authors:** Maria Eduarda Bertoni Trombeta, Ana Lídia Borges Figueiredo Rodrigues, Natan Rosa Rodrigues da Silva, Ana Clara Campagnolo Gonçalves Toledo, Victor Galli de Freitas, Edima de Souza Mattos

**Keywords:** eritropoietina, ensaios clínicos, hidroxiureia e anemia falciforme

## Abstract

**Introdução:**

A eritropoetina surge como uma potencial estratégia terapêutica em pacientes com anemia falciforme, devido à sua capacidade de modular a eritropoese e influenciar positivamente os processos anti-inflamatórios e antioxidantes. Objetivos: reunir informações e avaliar a qualidade metodológica de artigos quanto à eficácia da eritropoetina e da hidroxiureia no tratamento da hiper-hemólise em pacientes com anemia falciforme.

**Método:**

Revisão sistemática registrada na PROSPERO (CRD42024503662), conduzida segundo a diretriz PRISMA (). Bases de dados: MEDLINE, Embase, Web of Science, Cochrane Library, Scopus e CINAHL. Critérios de inclusão: portadores de anemia falciforme com hiper-hemólise pós-transfusional, submetidos a terapia combinada de eritropoetina e hidroxiureia em ensaios clínicos. As etapas – seleção dos estudos, extração de dados e qualidade do risco de viés – foram realizadas por dois autores independentes, usando as plataformas Rayyan QCRI e o formulário padronizado no Excel e a ferramenta ROB-2, Cochrane Library, respectivamente.

**Resultados:**

Dos 247 artigos inicialmente identificados, dois estudos foram incluídos na revisão. Furness . (2009) indicou benefício potencial da combinação no manejo pré-operatório da transfusão sanguínea, enquanto Goldberg (1990) mostrou melhora na saturação de oxigênio e na dor com o uso combinado. Ambos os estudos apresentaram alto risco de viés em diversos domínios metodológicos, incluindo geração da sequência aleatória, sigilo da alocação, cegamento e dados incompletos.

**Conclusão:**

A associação de hidroxiureia e eritropoetina sugere potenciais benefícios na redução de transfusões e melhora de alguns desfechos em pacientes com anemia falciforme. Entretanto, o alto risco de viés nos estudos incluídos limita a confiabilidade dos resultados. Novas pesquisas com delineamento robusto e controle rigoroso do viés são essenciais para confirmar a eficácia e a segurança dessa intervenção.

